# Complex network analysis of resting state EEG in amnestic mild cognitive impairment patients with type 2 diabetes

**DOI:** 10.3389/fncom.2015.00133

**Published:** 2015-10-29

**Authors:** Ke Zeng, Yinghua Wang, Gaoxiang Ouyang, Zhijie Bian, Lei Wang, Xiaoli Li

**Affiliations:** ^1^State Key Laboratory of Cognitive Neuroscience and Learning, IDG/McGovern Institute for Brain Research, Beijing Normal UniversityBeijing, China; ^2^Center for Collaboration and Innovation in Brain and Learning Sciences, Beijing Normal UniversityBeijing, China; ^3^Department of Vascular Neurosurgery, The Second Artillery General Hospital of PLABeijing, China; ^4^Department of Neurology, The Second Artillery General Hospital of PLABeijing, China

**Keywords:** resting state electroencephalography (rsEEG), amnestic mild cognitive impairment (aMCI), diabetes, phase lag index (PLI), graph analysis

## Abstract

**Purpose:** Diabetes is a great risk factor for dementia and mild cognitive impairment (MCI). This study investigates whether complex network-derived features in resting state EEG (rsEEG) could be applied as a biomarker to distinguish amnestic mild cognitive impairment (aMCI) from normal cognitive function in subjects with type 2 diabetes (T2D).

**Method:** In this study, EEG was recorded in 28 patients with T2D (16 aMCI patients and 12 controls) during a no-task eyes-closed resting state. Pair-wise synchronization of rsEEG signals were assessed in six frequency bands (delta, theta, lower alpha, upper alpha, beta, and gamma) using phase lag index (PLI) and grouped into long distance (intra- and inter-hemispheric) and short distance interactions. PLI-weighted connectivity networks were also constructed, and characterized by mean clustering coefficient and path length. The correlation of these features and Montreal Cognitive Assessment (MoCA) scores was assessed.

**Results:** Main findings of this study were as follows: (1) In comparison with controls, patients with aMCI had a significant decrease of global mean PLI in lower alpha, upper alpha, and beta bands. Lower functional connection at short and long intra-hemispheric distance mainly appeared on the left hemisphere. (2) In the lower alpha band, clustering coefficient was significantly lower in aMCI group, and the path length significantly increased. (3) Cognitive status measured by MoCA had a significant positive correlation with cluster coefficient and negative correlation with path length in lower alpha band.

**Conclusions:** The brain network of aMCI patients displayed a disconnection syndrome and a loss of small-world architecture. The correlation between cognitive states and network characteristics suggested that the more in deterioration of the diabetes patients' cognitive state, the less optimal the network organization become. Hence, the complex network-derived biomarkers based on EEG could be employed to track cognitive function of diabetic patients and provide a new diagnosis tool for aMCI.

## Introduction

Diabetes is a common metabolic disorder which can lead to chronic complications such as cardiovascular disease and peripheral neuropathy. The worldwide prevalence of diabetes has increased during the past five decades, with 382 million people with diabetes in 2013 (International Diabetes Federation, 2013). In diabetes, the Type 2 diabetes (T2D) caused by insulin resistance accounts for 90–95% of diabetes cases, which makes it an important public health issue. Several reports have indicated that T2D is associated with an increase in the risk of dementia and the proportion of patients who convert from mild cognitive impairment (MCI) to dementia (Koekkoek et al., 2015). MCI includes two subtypes: amnestic mild cognitive impairment (aMCI) and non-amnestic MCI, the former being considered to be a prodromal stage of Alzheimer's disease (AD) (Vos et al., 2013). Previous research showed that MCI progresses to AD at a rate of approximately 10–15% of patients per year (Wee et al., 2011). Moreover, results of prospective population-based studies showed that the proportion of MCI patients who convert to dementia is 1.5–3 times higher for those with diabetes compared with those without (Xu et al., 2010; Li et al., 2011). Currently no cure exists for AD, but administering certain medications in the early stages may delay the onset of symptoms (Alzheimer's Association, 2013). With the increase of the number of elderly patients with diabetes, it is becoming urgent and critical to explore methods to screen for the MCI patients with diabetes, so that the early interventions to these patients can be provided to reduce the likelihood of the conversion from MCI to AD.

Traditionally MCI is diagnosed based on clinical observations and cognitive test. But before clinical symptoms appear, the disease process is ongoing and damaging the brain. Therefore, early diagnosis of MCI is demanded. Lots of researches are engaged in the discovery and validation of potential biomarkers that allow early diagnosis and objective assessment of MCI. These biomarkers can be divided into two classes: measures of key proteins deposition and signs of neuronal injury (Albert et al., 2011). Biomarkers of proteins deposition include both positron-emission tomography (PET) evidence of β-amyloid deposition (Fagan et al., 2006) and cerebrospinal fluid (CSF) measures of increased total tau or phosphorylated-tau (p-tau) (Shaw et al., 2009). Biomarkers of neuronal injury reflect the neurodegeneration due to MCI, including brain atrophy and hypometabolism, obtained by magnetic resonance imaging (MRI) and PET (Jagust, 2006; Jack et al., 2008). However, the sensitivity and specificity of these aforementioned biomarkers are different for different international databases. Moreover, these measures have their limitations: PET are costly and expose patients to radiation; CSF are invasive; and MRI are relatively expensive for mass screening of the MCI population. Therefore, a non-invasive and cost-effective tool is urgently needed.

Recent studies have demonstrated that the cerebral EEG rhythms can reflect the underlying brain activity, and could be regarded as a potential diagnostic tool for MCI (Jackson and Snyder, 2008). Compared with other techniques, EEG offers a relatively inexpensive, noninvasive, potentially portable, and replicable method for assessing age-related and disease-related neurophysiologic changes. Studies have repeatedly observed two major effects of MCI on EEG (Dauwels et al., 2011): slowing of EEG (more low-frequency power) and reduced complexity. These changes in EEG data have been used as biomarkers to diagnose subjects with MCI. However, these features tend to vary across subjects, therefore have insufficient specificity (Gallego-Jutglà et al., 2015). One limitation is that these features only characterize abnormalities in local brain region, whereas the disorder of MCI is closely correlated with abnormal activity in multiple brain areas (Misra et al., 2009; Bai et al., 2012). Thus, a diagnosis method considering the whole brain activities would be more suitable, e.g., the brain network analysis, which reflects interactions between different brain regions rather than local dysfunction. In brain network analysis, the interactions between each pair of regions are quantified. This structure of interaction is often termed functional connectivity, which refers to measuring statistical synchronization between physiological signals (such as EEG) recorded from different brain regions (David et al., 2004; Pereda et al., 2005).

In brain network studies, it is very important to consider whether synchronization between signals recorded at different sensors can accurately reflect the physiological interactions between different brain regions (Wen et al., 2015). A very serious problem unique to EEG is that the active reference electrode will contribute similar components to EEG signals recorded at different electrodes and yield spurious synchronization. Xu et al. have shown how different reference electrode methods, including vertex reference, average reference, and zero reference, affect the diagnosis of MCI (Xu et al., 2014). Moreover, nearby EEG electrodes are likely to pick up activity of common sources, which give rise to strong synchronization between recorded signals that reflect simple volume conduction rather than true functional connectivity (Srinivasan et al., 2007). Two approaches have been proposed to overcome the aforementioned problems. One is to estimate synchronization between signals from reconstructed sources (“source space”) rather than the actually recorded signals (“signal space”). Though this method has some added benefits of dealing with interactions between anatomically well-defined brain regions, no unique way exists to reconstruct the corresponding sources (Hadjipapas et al., 2005). The other approach is to extract synchronization between signals which is not or at least unlikely due to volume conduction. The phase lag index (PLI), reflecting the consistency with which one signal is phase leading or lagging with respect to another signal, was introduced to assess the true interactions in this direction (Stam et al., 2007). Previous studies have shown that PLI was much less affected by the influence of volume conduction and active reference electrodes than traditional measures like coherence or the imaginary component of coherence (Stam et al., 2007). Based on the PLI to calculate the functional connection, this work attempts to investigate the abnormal functional connectivity of MCI, in view of global and local brain networks.

However, abnormalities of functional connectivity in MCI *per se* may not yet explain why the large scale brain networks are functioning abnormally. Connectivity studies in MCI are generally very descriptive and lack a more robust framework to discriminate normal and abnormal networks in the brain. In recent years, the graph theory has been introduced to study topographic organization of large scale brain networks (Reijneveld et al., 2007; Bullmore and Sporns, 2009). Graph theory provides complex network models of the brain to better understand the relation between network structure and undergoing processes. Particularly, Watts and Strogatz introduced so-called “small-world” networks, which have a relatively high amount of “local clustering,” meaning that nodes are often connected to their neighbors, combined with relatively short “path lengths,” meaning that from any node it takes just a few steps to reach any other node in the network (Watts and Strogatz, 1998). Small-world network considers both the quality of local information processing and the co-operation of distant brain regions. There are now accumulating evidences that both structural and functional brain networks in healthy subjects display a “small-world” type network organization characterized by a combination of high local clustering and short path length (Smit et al., 2008; Bullmore and Sporns, 2012). As for patients, several recent studies have revealed a loss of “small-worldness” of brain networks due to neurodegeneration (Stam, 2014). However, current studies mainly focus on the brain network of dementia patients, but few have analyzed small-world property in MCI patients. Furthermore, findings in the MCI patients are quite contradictory among studies, since some of them report no significant changes of brain network in MCI whereas others show decreased or increased “small-worldness.” Specifically, Seo et al. reported that local clustering of networks was lower in MCI compared to normal cognitive subjects (Seo et al., 2013), whereas Vecchio et al. found a significant increment of the clustering coefficient for MCI group (Vecchio et al., 2014). Besides, both the above two studies did not observe obvious difference in path length between two groups, whereas Xu et al. found that the MCI group had increased path length; using this network feature allows to distinguish the two groups with 90% accuracy (Xu et al., 2014). Hence, it is still uncertain that whether MCI individuals would exhibit a disrupted small-world property similar to those of dementia patients, and more work are needed to make clear this problem, especially for the MCI patient with diabetes.

In the present study, we intend to study in more detail the brain networks changes in aMCI with T2D. In particular, we employ resting state EEG (rsEEG) and attempt to address the following three questions:

1. Whether the aMCI in T2D have a specific loss of either short distance or long distance interactions between particular regions.
2. Whether brain networks of the aMCI in T2D are characterized by a loss of small-world architecture.
3. Is there a relationship between network architecture and general cognitive state in T2D?

To this end, EEG was recorded during an eyes-closed no-task state in 28 T2D patients including 16 subjects in aMCI and 12 subjects in normal cognitive state. The PLI as a measure of functional connectivity was computed between all channels of interest for signal filtered in delta, theta, lower alpha, upper alpha, beta, and gamma bands. The PLI values were averaged for short distance and long distance (intra- and interhemispheric) channel pairs to explore abnormality of functional connectivity. In addition, weighted graphs were built from functional connectivity to calculate the clustering index and path length to examine topographic changes of brain network. Finally, how the dynamic changes in global network architecture correlated with the generic cognitive status was analyzed.

## Materials and methods

### Participants

This study involved 28 T2D patients who satisfied the diagnosis criteria for diabetes (American Diabetes Association, 2013) in the Department of Neurology, General Hospital of Second Artillery Corps of PLA, Beijing, China. These participants were divided into 2 groups: aMCIs and controls. The following table (Table 1) reports information about the participants' age, diabetes duration, education level, generic cognitive status estimated by the Mini Mental State Examination (MMSE) and the Montreal Cognitive Assessment (MoCA) test, and the number of participants per group.

**Table 1:** **Mean age, diabetes duration, cognitive status and the number for the participants of each group enrolled in the present study**.

**Group**	**Number**	**Age (years)**	**Education level (years)**	**Diabetes duration (years)**	**MMSE**	**MoCA**
aMCI	16 (5 males)	69.7 ± 8.4	12.9 ± 1.8	9.3 ± 2.4	27.9 ± 0.5	22.4 ± 0.5
Control	12 (6 males)	73.3 ± 4.6	13.8 ± 3.0	14.0 ± 3.1	28.8 ± 0.2	27.0 ± 0.5

The study protocol has taken consent from the ethics committee of Beijing Normal University, and all patients gave written informed consent that their clinical data might be used and published for research purposes. The experiment was conducted in accordance with the Declaration of Rits (1964).

### Neuropsychological examination and inclusion criteria

In this study, besides MMSE and MoCA, the neuropsychological examination also included other tests aiming to assess the participant's generic cognitive status as well as specific cognitive domains (verbal memory, independent living, etc.). The details can be found in Bian et al. (2014).

All the selected aMCI patients with diabetes satisfied the following criteria (Petersen, 2004; Albert et al., 2011): (1) impaired cognitive function in one or several domains (such as memory), typically 1–1.5 standard deviation below normative data; (2) cognitive complaint usually coming from the patients or their family; (3) essentially preserving most activities of daily life; (4) not demented.

### EEG recording and pre-processing

Neurophysiological data were recorded while subjects were seated in a comfortable arm chair, using a high-density 128-channel EGI system of Net Amps 300 amplifiers [Electrical Geodesics Inc. (EGI), Eugene, OR]. The vertex sensor (Cz) was set as the reference electrode and the sampling rate was 1000 Hz. The impedances of all electrodes were kept below 50 kΩ, as recommended by EGI guidelines. During the recording, patients were instructed to keep relaxed, with their eyes closed, for at least 5 min.

**Figure 1:**
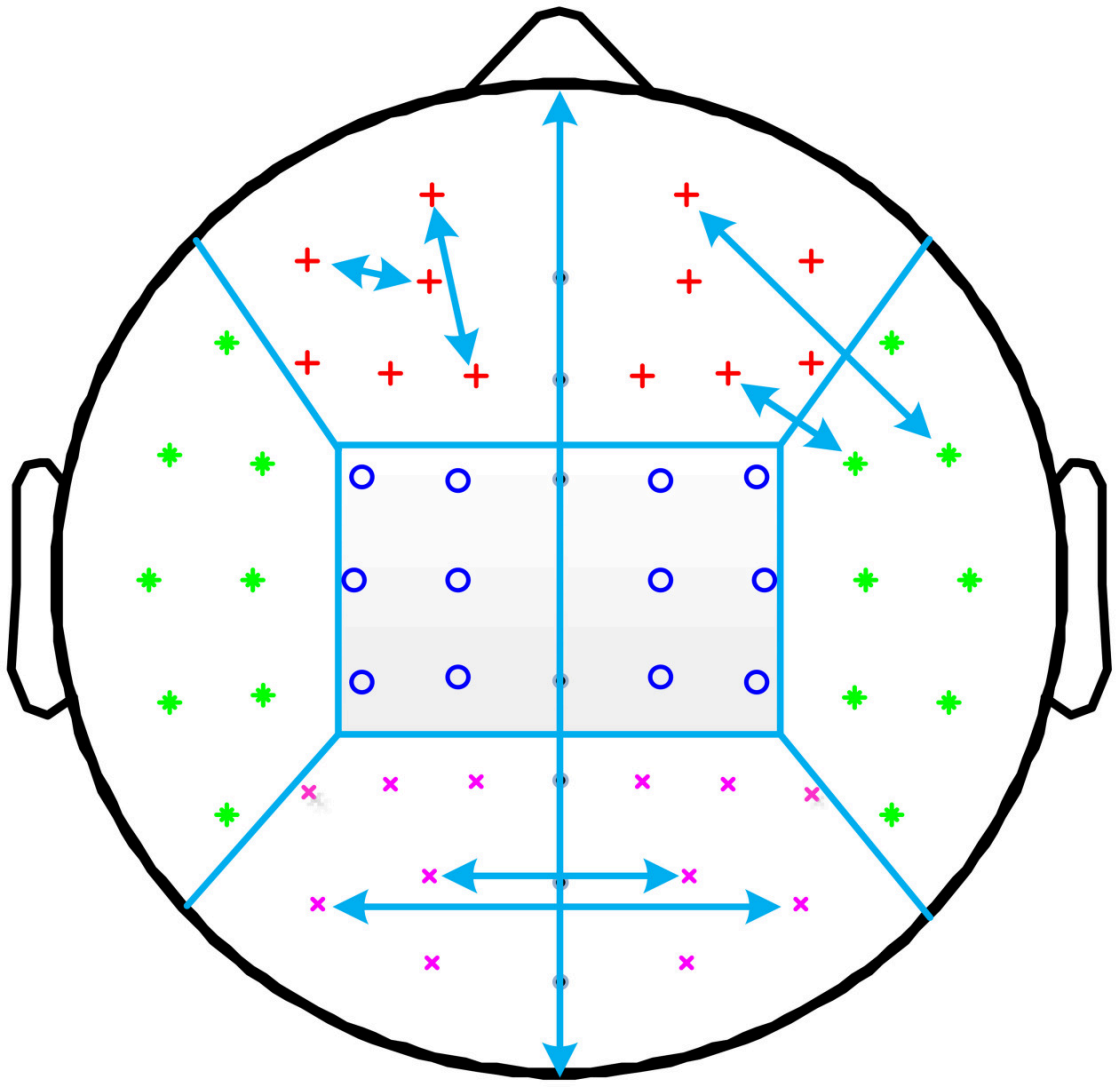
**Illustration of the allocation of channel pairs for short and long distances**. Channels are grouped into frontal (red plus sign), central (blue circle), occipital (purple cross), and temporal (green asterisk) regions for both hemispheres. The short distance PLI is computed as the average PLI between all channel pairs within the same region (two such pairs are shown for the left frontal region). Long distance PLI is computed from channel pairs where one channel is in one region, and the other channel is in another region. This is illustrated for right temporo-central long distance PLI and for occipital interhemispheric long distance PLI.

For further off-line processing, 59 electrodes were firstly selected according to the international 10-10 system, and the division of the brain regions is depicted in Figure 1. Then, the recorded EEG was re-referenced using the average of left and right mastoid sensors, and down-sampled to 250 Hz. A notch filter centered at 50 Hz was used to remove the line noise. Other artifact components were automatically rejected by combining ensemble empirical mode decomposition and independent component analysis (Zeng et al., in revision). After that, visual inspection was performed to further eliminate data segments contaminated by noise. Finally, for each subject, epochs of 3 min were segmented for further analysis. The aforementioned pre-processing procedure was performed using the MATLAB Signal Processing Toolbox and EEGLAB (Delorme and Makeig, 2004).

### PLI calculation

Functional connectivity between different brain regions was computed with the PLI (Stam et al., 2007). The PLI is a measure of the asymmetry of the distribution of phase differences between two signals. It reflects the consistency with which one signal is phase leading or lagging to another signal. In short, if the phase differences between two time series are △ϕ(*t*_*k*_)(*k* = 1 …*N*), PLI can be computed by

$$\begin{array}{l} \text{PLI}=|\langle \text{sign}\left[△\phi \left(t_{k}\right)\right]\rangle | \end{array}$$

where 〈·〉 is the mean value operator. The PLI ranges between 0 and 1. A PLI of zero indicates either no coupling or coupling with a phase difference centered around 0 mod π. And a PLI of 1 indicates perfect phase locking at a value of △ϕ different from 0 mod π. The stronger the nonzero phase locking is, the larger PLI will be.

PLI was computed for the following frequency bands: delta (1–4 Hz), theta (4–8 Hz), lower alpha (8–10 Hz), upper alpha (10–13 Hz), beta (13–30 Hz), and gamma (30–45 Hz). For each frequency band, the result of PLI for all pair-wise combinations of channels is an *N*×*N* synchronization matrix, *N* = 59, in which each entry PLI_*i, j*_ contains the value of the PLI for the channels *i* and *j*.

Besides a global mean PLI calculation, further averaging was done to obtain long distance intra- and interhemispheric and short distance local measures. For this analysis, EEG channels were grouped into four regions (frontal, temporal, central, and occipital) for each hemisphere. Long distances (12 intra-hemispheric: fronto-temporal, fronto-central, fronto-occipital, temporo-central, occipito-temporal, occipito-central; 4 interhemispheric: frontal, central, occipital, and temporal) involved synchronizations between two different regions (within one hemisphere or homolog regions of two hemispheres), and short distances involved synchronizations within one region. Midline channels were not used. The allocations of channel pairs for short and long distances are illustrated in Figure 1.

### Graph analysis

In this work, graph analysis was adopted to explore the difference between the two groups in the brain network. The nodes in the graph are represented by channels and the edges are defined as the synchronization between two EEG signals recorded at the corresponding channels. In this study, the aforementioned PLI values were assigned to corresponding edges to reflect strength between the two nodes. For each subject in each frequency band, we defined a network of 59 nodes and the corresponding edge weights mapping from PLI matrix. Weighted graph was directly used to analyze the brain network to avoid choosing an arbitrary threshold for binary graph analysis.

Graphs can be characterized by many measures. Two fundamental measures are the clustering coefficient and path length, both of which have been widely used to analyze the brain function network. In short, the clustering coefficient for a node generally represents the proportion of its neighboring nodes that are connected among each other. The weighted clustering coefficient of vertex *i* is defined as

$$\begin{array}{l} C_{i}=\frac{\sum_{k\neq i}\sum_{l\neq i,l\neq k}w_{ik}w_{il}w_{kl}}{\sum_{k\neq i}\sum_{l\neq i,l\neq k}w_{ik}w_{il}} \end{array}$$

where *w* is the edge weight between two nodes. And the mean clustering coefficient of the whole network is defined as:

$$\begin{array}{l} \text{C}=\frac{1}{N}\overset{N}{\underset{i=1}{\sum }}C_{i} \end{array}$$

The path length for weighted graph was calculated according to the approach of Latora and Marchiori (2001). The length of an edge is defined as the inverse of the weight, i.e., *L*_*ij*_ = 1/*w*_ij_ if *w*_*ij*_ ≠ 0, and *L*_*ij*_ = ∞ if *w*_*ij*_ = 0. The shortest path between two nodes is the path with the shortest length between the two nodes. The average weighted path length of the entire graph is estimated as:

$$\text{L}=\frac{1}{\frac{1}{N\left(N-1\right)}\sum_{i\;=\;1}^{N}\sum_{j\;\neq \;i}^{N}\left(1/L_{ij}\right)}$$

### Statistical analysis

In this study, ANOVA was used to analysis the difference in the subject characteristics. And group differences in PLI distribution were tested with two-tailed *t*-tests for independent samples (not assuming equal variance). As graph measures showed a non-Gaussian distribution, group differences were tested with Mann–Whitney *U*-tests for independent samples. Associations between general cognitive states and network-derived measures were assessed with Pearson's linear correlations. A significance level of *p* < 0.05 was used.

## Results

### Subject characteristics

The two groups were matched in age, diabetes duration and education level, but not in gender. Nevertheless, no effect of gender distribution in the two groups on PLI and network-derived measures was found. There were significant differences in scores of MoCA between aMCI and controls (*p* < 0.001). The scores of MMSE were lower in aMCI than controls, while these differences were not statistically significant. Hence, we only focused on MoCA in the following study.

### PLI analysis

PLI was computed for all pair-wise combinations of channels in six frequency bands respectively. Group differences in mean PLI for each frequency band between the aMCI and controls were tested with two-tailed *t*-tests for independent samples.

**Figure 2:**
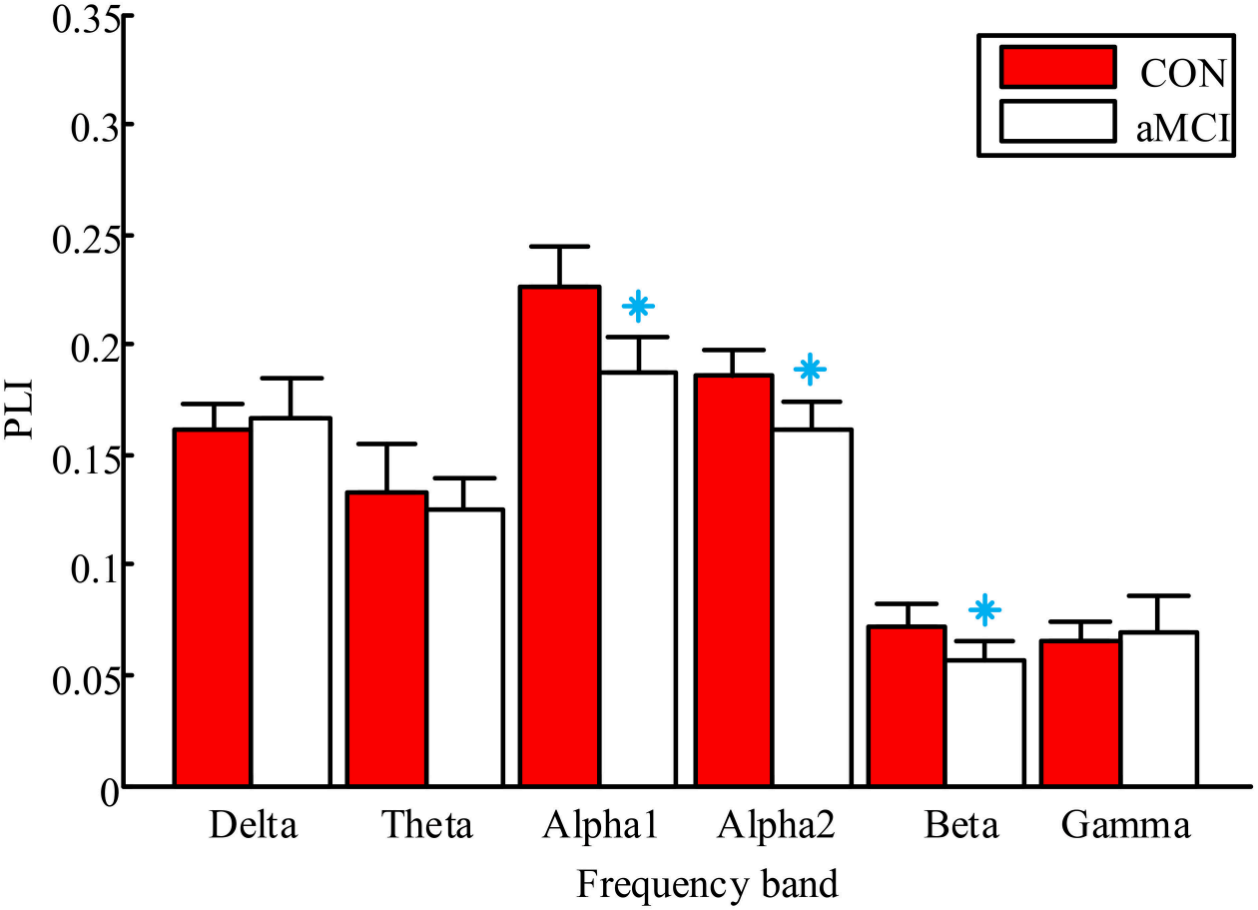
**Mean PLI averaged over all pairs of EEG channels for aMCI and controls in six frequency bands**. The control group and aMCI group are represented by red and white boxes respectively. Error bars are standard deviations. Significant differences between aMCI and controls with two-tailed *t*-test (*P* < 0.05) are presented by blue asterisks.

The results of group differences in global mean PLI are shown in Figure 2. For convenience, the lower alpha and upper alpha are labeled alpha1 and alpha2 in the figure, respectively. The mean PLI was significantly lower in the aMCI group in the low alpha band (*p* = 0.031), high alpha band (*p* = 0.028) and beta band (*p* = 0.043). There were no significant differences in other frequency bands.

**Figure 3:**
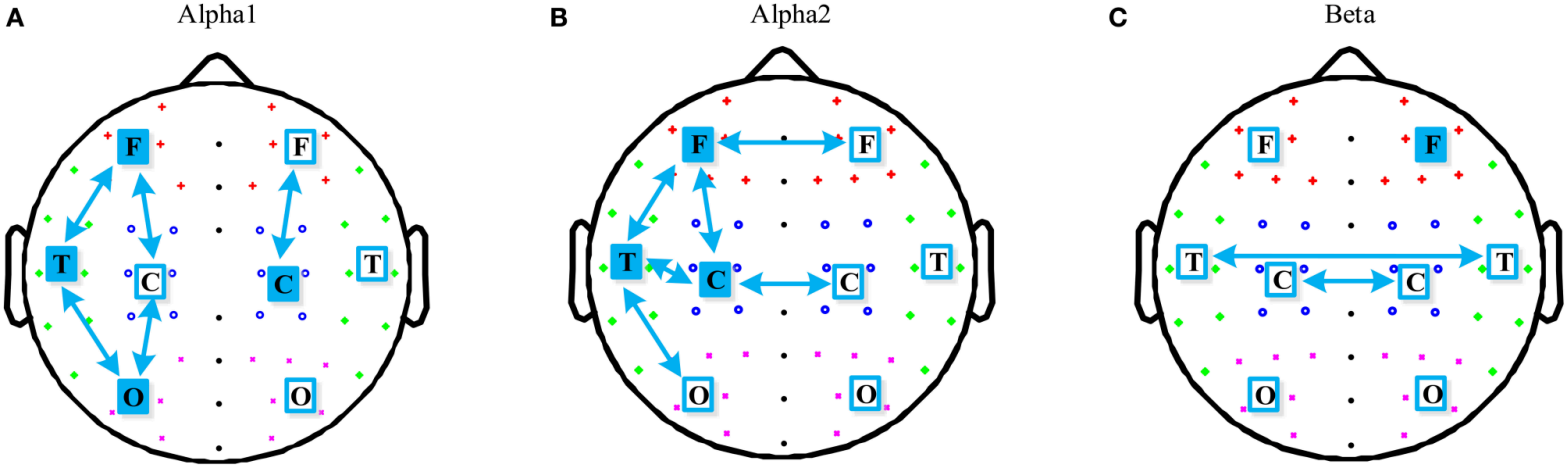
**Schematic illustration of significant differences in region mean PLI for short distance (indicated by filled squares) and long distance (indicated by arrows) in the alpha1, alpha2, and beta bands**. And the region differences between the two groups in the three frequency bands are shown in **(A)**, **(B)**, and **(C)** respectively.

The region differences between the two groups in the alpha1, alpha2, and beta band were further investigated and are illustrated in Figures 3A–C respectively. For the alpha1 band, aMCI group had significant lower PLI in left fronto-temporal (*p* = 0.015), fronto-central (*p* = 0.017), occipito-temporal (*p* = 0.006), occipito-central (*p* = 0.005), and right fronto-central (*p* = 0.039). Mean PLI in local left frontal (*p* = 0.022), temporal (*p* = 0.025), occipital (*p* = 0.021), and right central (*p* = 0.028) were also decreased in the aMCI group. For the alpha2 band, aMCI group showed a PLI decrease in local left frontal (*p* = 0.017), temporal (*p* = 0.018), and central (0.023). All the region differences in intra-hemispheric long distance concentrated on the left hemisphere: fronto-temporal (*p* = 0.029), fronto-central (*p* = 0.024), temporo-central (*p* = 0.018), and occipito-temporal (*p* = 0.024). Besides, mean PLI in interhemispheric temporal (*p* = 0.023) and central (*p* = 0.038) were also decreased in the aMCI group. For the beta band, aMCI group had significant lower PLI in local right frontal (*p* = 0.043), interhemispheric temporal (*p* = 0.025), and central (*p* = 0.028).

### Graph analysis

Clustering coefficient and path length of brain function network were calculated for both aMCI and control groups in each frequency band. The non-parametric Mann–Whitney *U*-test was used to test the group differences in network characteristics.

**Figure 4:**
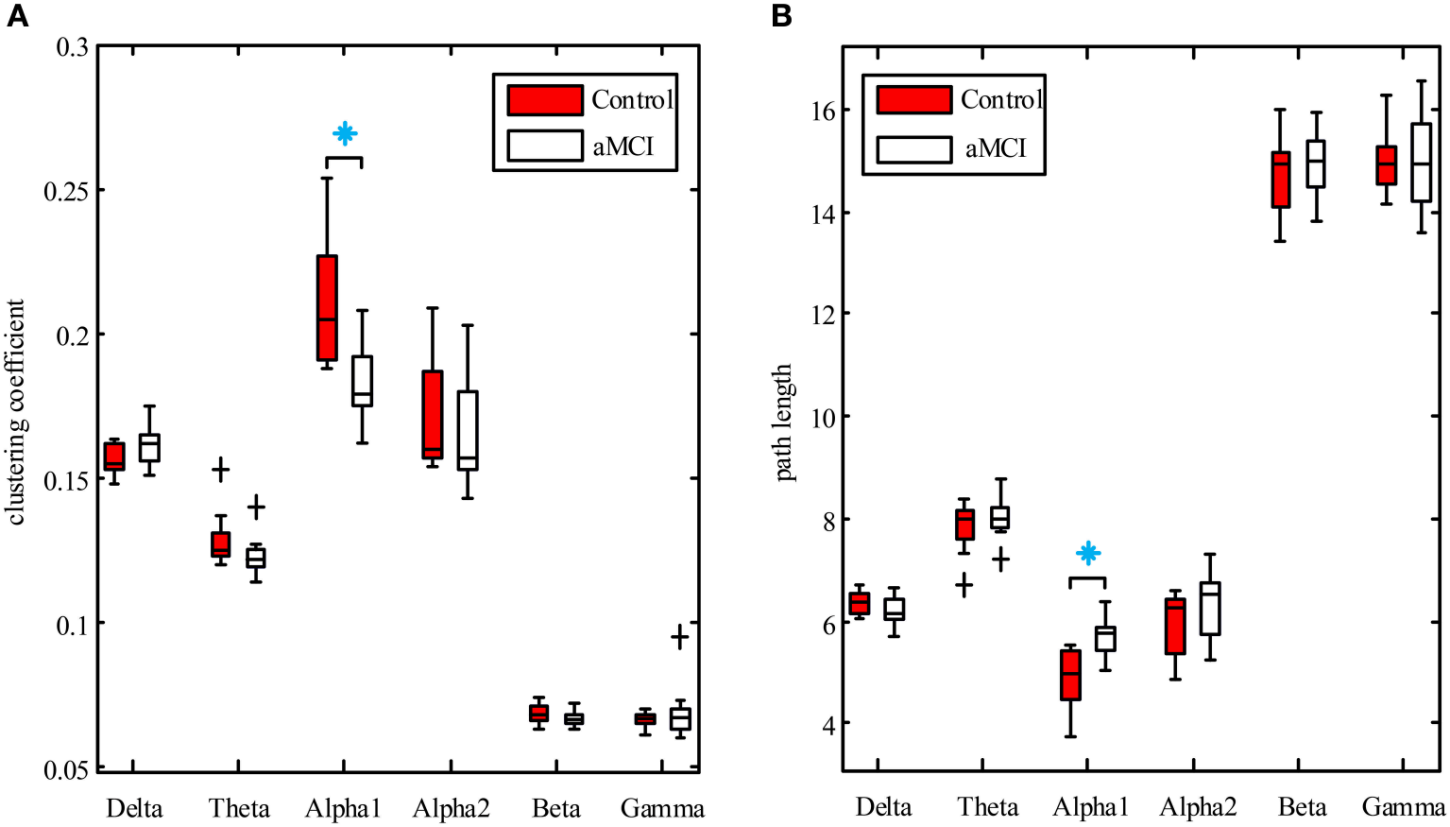
**Boxplots of clustering coefficient (A) and path length (B) for control (red box) and aMCI (white box) groups in each frequency band**. Boxplots show median value, interquartile range, extremes, and outliers (black plus). Significant differences between aMCI and controls with Mann–Whitney *U*-test (*P* < 0.05) are presented by blue asterisks.

Boxplots of clustering coefficient and path length for the two groups in each frequency are shown in Figures 4A,B, respectively. The results revealed that clustering coefficient was significantly lower in aMCI group compared to controls in the lower alpha band (*U* = 117, *p* = 0.025), but not in the other bands. The path length of the aMCI group increased in the low alpha band (*U* = 87, *p* = 0.04), while no differences between the two groups in other bands were found.

### Correlation between cognitive status and network characteristics

**Figure 5:**
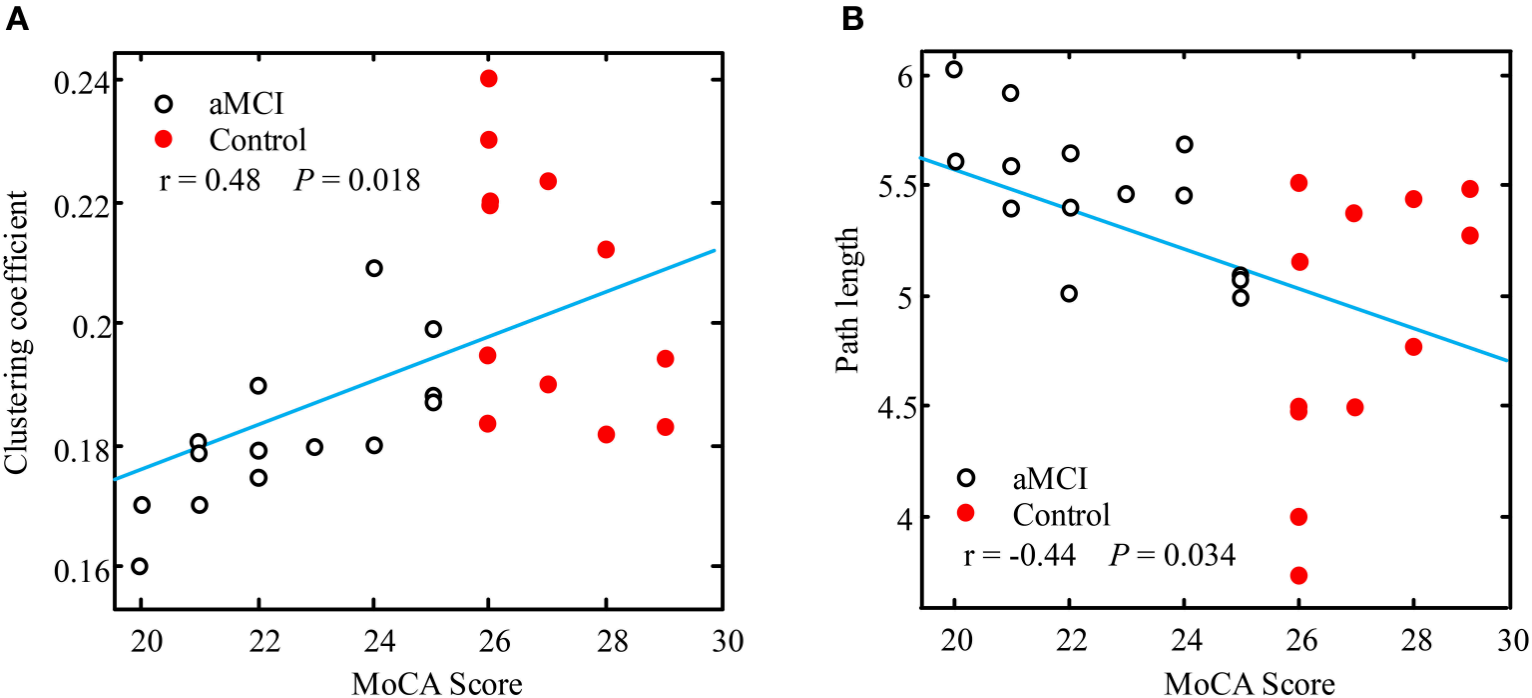
**Visualization of the linear correlation between network characteristics and MoCA score**. The positive correlation of clustering coefficient with MoCA in the lower alpha band is shown in **(A)** (*r* = 0.48, *p* = 0.018), and the negative correlation of path length with MoCA in the low alpha band is shown in **(B)** (*r* = −0.44, *p* = 0.034). aMCI and controls group were combined for this analysis.

In order to test if there is correlation between cognitive status and the network characteristics, Pearson's linear correlations were computed for all subjects (aMCI and controls) put together in one group. Figure 5 illustrates these correlations through scatter plots of the MoCA data distributions against the clustering coefficient and path length values respectively. The results showed a significant positive correlation between MoCA and clustering coefficient (*r* = 0.48, *p* = 0.018) (shown in Figure 5A), and a significant negative correlation between MoCA and path length at the low alpha band (*r* = −0.44, *p* = 0.034) (shown in Figure 5B). Based on the significant correlation of network architecture and the cognitive status, this finding may suggest that the degree of network performance can reflect the deficiency in generic cognitive processing.

## Discussion

T2D may increase the risk of cognitive impairment and accelerate the progress from MCI to dementia. We attempted to explore the changes in brain functional network to distinguish aMCI from controls in patients having T2D. We found that resting-state functional connectivity of EEG is decreased in aMCI patients in the lower alpha, upper alpha, and beta bands. This finding supports the concept of aMCI as a disconnection syndrome. Furthermore, changes in functional connectivity in aMCI patients did not involve the whole brain regions to the same extent, which suggested a heterogeneous disruption of functional network structure. This idea was confirmed by graph analysis of the organization of brain network, which revealed lower clustering coefficient and higher path length in aMCI at the lower alpha band. The changes suggest a loss of “small-worldness” of brain networks in aMCI than in control subjects. In addition, we also found a statistically significant correlation between network-derived measures and cognitive status as measured with MoCA, which suggested that network characteristics can reflect the process of cognitive deterioration in patients with T2D.

The resting-state EEG was used in this work to analyze the brain network in the absence of task performance or sensory stimulation. These measurements can identify abnormalities in MCI, whereas the most widely used evoked potential is not well suited (Fox and Greicius, 2010). In task-based evoked potential studies, only time-locked neural responses to events of interest were studied, and all other spontaneous activity are considered background noise. So task-dependent changes in brain network are difficult to interpret the functional differences in individuals with MCI at rest (Fox et al., 2006). Furthermore, lots of studies have suggested that the brain system operates with intrinsic resting-state integration and external sensory information only interacts with, rather than determines, the operation of brain system (Fox and Raichle, 2007; Raichle and Snyder, 2007). In addition, there are also several practical advantages of using rsEEG to study abnormal brain function in neural disorders. Resting-state approaches don't require subjects to make responses for some event, which is particularly promising for studying more severely impaired and/or younger patients who may not be able to perform tasks accurately because of cognitive, physical, or developmental challenges. EEG of resting-state is also a promising tool to monitor the evolution of the disease and the effect of treatment (Zeng et al., 2015).

Despite lots of advantages in the understanding of functional interactions among brain regions, EEG still faces the problem of volume conduction, which may give rise to spurious interactions. In the present study, we adopt PLI to describe the interaction and group the electrode pairs in short and long distances. While PLI estimated in this way will be influenced by volume conduction, it is less likely that volume conduction can explain group difference in PLI between MCI and controls. Furthermore, several of abnormal connections involve in long distance, which are less likely to be due to volume conduction. Although Peraza et al. has demonstrated that the PLI would overestimate the small-worldness due to volume conduction, they also showed that brain network based on the PLI was closer to the idea network relative to the network based on the coherence and phase synchrony (Peraza et al., 2012). Hence, PLI is still a good choice to apply for brain network inference from EEG recordings, at least to a large degree. The choice of the type of graph to analyze brain network also can lead to bias. Generally, there are two types of undirected graphs: weighted graph and binary graph. For the latter, some threshold should be selected to transform the synchronization matrix into its adjacency matrix, the characteristics of the binary graph is therefore crucially depends on the selected threshold (Zanin et al., 2012). To avoid artificial arbitrariness, the present work directly adopts the weighted graph to analyze the brain network to get comparable results.

The pattern of global functional connectivity changes in the current study shows similarities as well as differences with previous EEG and MEG studies. A lower level of global synchronization in MCI was presented in alpha and beta bands, which has been reported by early studies (Pijnenburg et al., 2004; Koenig et al., 2005; López et al., 2014; Wen et al., 2014). However, many other studies with MCI patient also demonstrated different abnormalities of functional connectivity. Specifically, Stam et al. found that the synchronization measured by synchronization likelihood was similar in controls' EEG and those of MCI patients (Stam et al., 2003), whereas Gomez et al. showed that functional connectivity measured by coherence was lower in the MCI group than control group at all frequency bands (Gómez et al., 2009). And Lopez reported that compared with controls, MCIs showed lower phase locking values in alpha, beta, and gamma bands in resting state while higher phase locking values in delta, theta, and gamma bands during cognitive tasks (López et al., 2014). The differences are probably due to the different condition in EEG recording (task or resting conditions) or the method to calculate functional connection. Nevertheless, our results are generally consistent with most studies on loss of average synchrony in rest condition of MCI patient. This decrease in synchrony is often attributed to a functional disconnection of the neocortex. Therefore, this study suggests that the decreased mean functional connection can be as a sensitive index to distinguish aMCI from subjects with normal cognitive function.

To reveal the abnormal functional connection further, the present study also investigated the relative contribution to the impaired functional connectivity in MCI from short distance and long distance interactions in lower alpha, upper alpha, and beta band. Lower short and long intra-hemispheric functional connections in both lower alpha and upper alpha band mainly distributed at the left hemisphere, especially in the temporo-fronto-central cortices. Similar results were also reported in previous studies (Stam et al., 2006, 2009). These findings were consistent with the current neurological knowledge of the trend of AD progression, that early changes are seen in left hemisphere followed by right hemisphere areas (Sankari et al., 2011). Moreover, both the upper alpha and beta bands showed a loss of long distance interhemispheric functional connectivity, where the former showed in frontal and temporal regions, and the latter showed in temporal and occipital regions. Though combining MRI and electroencephalography, it has been reported that lower interhemispheric functional connection in MCI was correlated with atrophy of the corpus callosum (Pogarell et al., 2005). Our results further support the concept to some extent. Besides, the current work revealed that left temporal region was most involved in the abnormal functional connection. Other studies in MCI and AD have also stressed the importance of left temporal region and suggested that left temporal disturbance have been associated with a higher chance of conversion to MCI (Maestú et al., 2005, 2006).

Aided by the advanced graph theoretical approaches, we investigated changes in functional network organization of MCI by clustering coefficient and path length, which were two different perspectives of information segregation and integration. The result of present study showed that clustering coefficient was decreased and path length was increased in the lower alpha band. This suggested that MCI were associated with disrupted segregation and integration in brain networks. Furthermore, according to the definition of small-world network, it represented an optimal organization in terms of local specialization (large clustering) and global integration (small path length). Hence, a lower cluster coefficient with longer path length meant a less optimal network organization and a loss of small-world network characteristics in MCI. Such kind of the network deterioration in MCI also has been reported by others using EEG (Frantzidis et al., 2014), MEG (Buldú et al., 2011), and functional MRI (Yao et al., 2010). Considering the random rewiring model in Watts and Strogatz (1998), the functional network in MCI was characterized by a shift from organized small-world networks to random network. The trend to random network in MCI may be a final common pathway for different types of brain damage (such as multiple sclerosis, traumatic brain injury, and epilepsy), resulting from loss of neurons and connections as well a random outgrowth of new connections (Stam, 2014). Therefore, the graph techniques could be a promising approach to study the underlying mechanisms in MCI.

In addition, this study found that cognitive status measured by MoCA has significantly positive correlation with cluster coefficient and negative correlation with path length in lower alpha band. That is, the more deterioration of cognitive states the patients had, the less optimal the network organization was. This finding was in agreement with a previous finding of a positive correlation between small-worldness and MoCA scores in a group of MCI and AD patients (Frantzidis et al., 2014). A longitudinal EEG study also found that the progression of AD is characterized by a reduction of efficiency and a loss of small-worldness of the pathological brain (Morabito et al., 2015). These correlations confirmed the feasibility and value of advanced graph theory approaches in EEG to study aMCI in diabetes, and provide quantitative predictions of changes in brain network as disease progresses.

This study showed that the network analysis of rsEEG may provide an efficient method to monitor the cortical dysfunction associated with the cognitive decline of diabetic patients. The network-derived measures may eventually play a role in early detecting aMCI or in guiding diagnosis of aMCI in diabetes. Hence, early intervention can be carried out to slow the development pace of aMCI to AD. However, these results are still limited. Larger prospective studies are necessary to verify the findings in this study.

## Author contributions

The work presented here was carried out in collaboration between all authors. KZ, OY, and XL designed the research; ZB and LW acquired and interpreted the data for this research; KZ and YW analyzed the data; KZ, YW, and XL wrote the paper. All authors have read and approved the final published manuscript.

### Conflict of interest statement

The authors declare that the research was conducted in the absence of any commercial or financial relationships that could be construed as a potential conflict of interest.
